# Human-to-Cattle *Mycobacterium tuberculosis* Complex Transmission in the United States

**DOI:** 10.3389/fvets.2021.691192

**Published:** 2021-07-12

**Authors:** Jason E. Lombard, Elisabeth A. Patton, Suzanne N. Gibbons-Burgener, Rachel F. Klos, Julie L. Tans-Kersten, Beth W. Carlson, Susan J. Keller, Delora J. Pritschet, Susan Rollo, Tracey V. Dutcher, Cris A. Young, William C. Hench, Tyler C. Thacker, Claudia Perea, Aaron D. Lehmkuhl, Suelee Robbe-Austerman

**Affiliations:** ^1^United States Department of Agriculture: Animal and Plant Health Inspection Service, Veterinary Services, Field Epidemiologic Investigation Services, Fort Collins, CO, United States; ^2^Wisconsin Department of Agriculture, Trade and Consumer Protection, Madison, WI, United States; ^3^Wisconsin Department of Health Services, Division of Public Health, Madison, WI, United States; ^4^North Dakota Department of Agriculture, State Board of Animal Health, Bismarck, ND, United States; ^5^North Dakota Department of Health, Bismarck, ND, United States; ^6^Texas Animal Health Commission, Austin, TX, United States; ^7^United States Department of Agriculture: Animal and Plant Health Inspection Service, Veterinary Services, Ruminant Health Center, Fort Collins, CO, United States; ^8^United States Department of Agriculture: Animal and Plant Health Inspection Service, Veterinary Services, National Veterinary Services Laboratories, Ames, IA, United States

**Keywords:** *Bovine tuberculosis*, zoonotic disease, human-to-cattle transmission, public health, dairy employees

## Abstract

The *Mycobacterium tuberculosis* complex (MTBC) species includes both *M. tuberculosis*, the primary cause of human tuberculosis (TB), and *M. bovis*, the primary cause of bovine tuberculosis (bTB), as well as other closely related *Mycobacterium* species. Zoonotic transmission of *M. bovis* from cattle to humans was recognized more than a century ago, but transmission of MTBC species from humans to cattle is less often recognized. Within the last decade, multiple published reports from around the world describe human-to-cattle transmission of MTBC. Three probable cases of human-to-cattle MTBC transmission have occurred in the United States since 2013. In the first case, detection of active TB disease (*M. bovis)* in a dairy employee in North Dakota prompted testing and ultimate detection of bTB infection in the dairy herd. Whole genome sequencing (WGS) demonstrated a match between the bTB strain in the employee and an infected cow. North Dakota animal and public health officials concluded that the employee's infection was the most likely source of disease introduction in the dairy. The second case involved a Wisconsin dairy herd with an employee diagnosed with TB disease in 2015. Subsequently, the herd was tested twice with no disease detected. Three years later, a cow originating from this herd was detected with bTB at slaughter. The strain in the slaughter case matched that of the past employee based on WGS. The third case was a 4-month-old heifer calf born in New Mexico and transported to Texas. The calf was TB tested per Texas entry requirements and found to have *M. tuberculosis*. Humans are the suspected source of *M. tuberculosis* in cattle; however, public health authorities were not able to identify an infected human associated with the cattle operation. These three cases provide strong evidence of human-to-cattle transmission of MTBC organisms and highlight human infection as a potential source of introduction of MTBC into dairy herds in the United States. To better understand and address the issue, a multisectoral One Health approach is needed, where industry, public health, and animal health work together to better understand the epidemiology and identify preventive measures to protect human and animal health.

## Introduction

The primary causative agents of human and bovine tuberculosis (bTB) in North America, *Mycobacterium tuberculosis* and *Mycobacterium bovis*, respectively, are included in the *Mycobacterium tuberculosis* complex (MTBC). Other members of the MTBC include *Mycobacterium orygis, Mycobacterium caprae, Mycobacterium microti, Mycobacterium pinnipedii, Mycobacterium mungi*, and *Mycobacterium suricattae* (1). The MTBC species are so closely related that they are now considered a single species, *M. tuberculosis*, with variants (1, 2).

Worldwide, tuberculosis is the leading cause of human deaths by any single infectious agent and responsible for approximately 1.2 M deaths in HIV-negative people in 2019 (3). Tuberculosis was a leading cause of human morbidity and mortality in the United States at the beginning of the twentieth century. A study conducted in 1912 reported that 66% of New York children diagnosed with tuberculosis (TB) in 1910 were infected with *M. bovis* (4). Based on the transmission of *M. bovis* to children through milk, multiple jurisdictions enacted laws requiring pasteurization. Cincinnati was the first city to establish pasteurization requirements in 1897; New York City followed in 1898 (5). Michigan became the first state to require pasteurization of milk in 1948 and all states have since followed suit. In addition to milk, meat from *M. bovis*-affected cattle is also a potential risk for human infection. Accordingly, the Federal Meat Inspection Act of 1906 (6, 7) gave the U.S. Department of Agriculture (USDA) the authority to inspect cattle before, during, and after slaughter as another tool for preventing zoonotic transmission of *M. bovis*. Lesions detected during this inspection, or entire carcasses if necessary, could be removed from the food chain. Not only did this reduce the risk of human infection, but also cattle with bTB could be identified and trace-back investigations to the herd of origin allowed for identification of bTB-infected herds. Slaughter inspection is the primary means of bTB surveillance in the United States today.

In addition to implementing these two mitigation strategies to reduce human exposure to *M. bovis*, state and federal authorities designed and implemented the U.S. Cooperative State-Federal Bovine TB Eradication Program to reduce the prevalence in cattle populations. The program officially began in 1917; at that time, approximately 1 in 20 cattle were infected with *M. bovis* (8). Reviews of the program and its progress have been published (9–12). Current estimates of animal- and herd-level prevalence of *M. bovis* in the United States are <0.002 and <0.006%, respectively (12). The partnership between State and Federal Animal Health Officials to conduct testing, share data, and conduct disease investigations has been critical to these advancements. In other parts of the world, including parts of Mexico and Central and South America, *M. bovis* infection rates in cattle and other hoof stock are much higher and serve as an important source for human disease (13). Additionally, consumption of unpasteurized dairy products remains a primary cause of human infections with *M. bovis* in North America (14–16).

Despite the early success of the program in the United States, the number of newly identified bTB-affected herds each year has remained relatively steady for the past 30 years (17). The advent of whole genome sequencing (WGS) has markedly advanced our ability to link sources of infection based on genetic similarities. For example, in Michigan WGS routinely supports wildlife as a source of infection. However, for cases outside of Michigan, even after extensive epidemiological investigations, including WGS and trace-back of purchased cattle, the source of disease and method of introduction into a herd is determined only about 40% of the time (17). Without a source of infection, risk mitigation, and disease eradication remain elusive.

Humans are considered the primary host for *M. tuberculosis* and animals are considered accidental hosts (18, 19). Human-to-animal transmission of MTBC organisms, primarily *M. tuberculosis*, is well-documented in many countries around the world. Many of these reports confirm finding *M. tuberculosis* in tissues and fluids from cattle (20–32) and also other animals, including dogs (33), non-human primates (34), elephants (35, 36), and parrots (37, 38). Most reports of cattle with *M. tuberculosis* are from countries where human *M. tuberculosis* prevalence is very high and likely to have been the source of introduction into the cattle populations.

With the technology now available to obtain DNA sequences of *M. bovis* isolates found in U.S. cattle, it is easier to discern the origin of certain *M. bovis* strains. This information may provide possible modes of transmission based on the most common ancestor in the genomic database and similarity of DNA sequences over time. Sharing isolate DNA sequence data between animal and public health will further our understanding of the complex transmission of MTBC organisms between humans and animals.

The objectives of this paper are to present the identified cases of human-to-cattle MTBC transmission in the United States and to highlight the importance of a multisectoral One Health approach in detecting and addressing human-to-cattle transmission of these important human and animal pathogens.

## Materials and Methods

### Case Selection

Records from the 174 bTB affected livestock herds identified in the USA between 1998 and 2020 were reviewed. As part of the State Federal Cooperative Bovine Tuberculosis program, each herd had been extensively investigated under that cooperative umbrella and if possible, the likely source of the infection was identified and investigated. Also included in this review were 422 records for tuberculous confirmed animals between 2001 and 2020 not associated with a herd, such as feedlot and dairy heifer development facilities.

Inclusion criteria were as follows: All *Mycobacterium tuberculosis* cases detected in livestock; and zoonotic tuberculosis (all were *M. bovis*) cases where humans associated with livestock were identified with active tuberculosis prior to the detection within the herd.

### Public Health Investigations

Public health authorities in the U.S. investigate all reported cases of TB disease in humans to ensure patients quarantine until non-infectious and receive and complete appropriate antimicrobial treatment. Contact investigations are routinely performed when patients have pulmonary TB and are considered infectious. Close contacts are screened for exposure risk and tested to determine their TB infection and disease status. The public health investigation may identify animals (especially livestock and captive wildlife) with an epidemiological link to the infectious patient. In North Dakota and Wisconsin, the public health agency alerts their animal health partners and a One Health investigation may be deemed necessary. All information on the human cases contained in this paper was collected during the normal process that occurs in these states when humans with TB are detected.

### Phylogenetic Analysis

Whole genome sequences were obtained from both animal health and public health investigations. NVSL's in-house vSNP pipeline was used for the analysis (see https://github.com/USDA-VS/vSNP). vSNP is a reference based, two-step pipeline. Briefly in step one, sequences were aligned to a reference; those identified as *M. bovis* were aligned to the reference genome AF2122/97 (GenBank accession NC_002945.4), and those identified as *M. tuberculosis* were aligned to the reference genome H37Rv (GenBank accession NC_000962.3). The alignment was performed using Burrows-Wheeler Aligner (BWA) (39) and SNPs were called using Freebayes (40). The variant call format (vcf) files created in step one were then added to a database of vcf files and step two was initiated which filters or flags unreliable and low quality variant calls, as well as groups sequences into user defined clades according to relatedness by identifying common SNPs. For each user defined group, step two outputs SNP tables in Excel, an aligned FASTA file and phylogenetic trees constructed with RAxML (41) using the aligned whole-genome SNP sequences under a GTR-CAT model of substitution and a maximum-likelihood algorithm. The annotated and position referenced SNP tables allow for quick error identification and correction. The trees were then manually compared to the SNP table to ensure accuracy of the model.

Tree visualization, annotation, and editing was performed with FigTree (http://tree.bio.ed.ac.uk/software/figtree/) and iTOL (42). Supplementary File 1 lists the accession numbers for the publicly available sequences from previous studies (17, 43, 44).

## Results

Three cases met the criteria for inclusion, two M. *bovis* affected dairy herds, and one *M. tuberculosis* infected 4-month-old calf at a dairy heifer development facility. Those cases are described in detail below.

### Case 1: North Dakota Dairy Herd

In October 2013, the North Dakota Department of Agriculture (NDDA), State Board of Animal Health was notified by the North Dakota Department of Health (NDDOH) that an employee at a North Dakota dairy was diagnosed with pulmonary TB with cavitary lung lesions. The case-patient, a man born in Mexico, had been recently diagnosed with another medical condition that likely suppressed his immune system resulting in active TB disease. The case-patient worked for the dairy for at least 3 years prior to his diagnosis. During this time, he worked for 9 consecutive months then returned to Mexico for the remaining 3 months each year. During his employment, the case-patient worked with all ages of dairy cattle. NDDOH tested three household contacts to the case-patient. All were latently infected with TB (LTBI) and were treated prophylactically with a 4-month course of antibiotics per CDC guidelines to prevent future disease.

The North Dakota operation housed 400 dairy cattle and 160 beef cattle. Beef and dairy heifers were often commingled for a few months each year. While the operation had no record of TB skin testing in the dairy or beef herds in the recent past, cull dairy and beef cattle from this operation were slaughtered at abattoirs with high granuloma submission rates for TB surveillance.

After meeting with the NDDOH's TB controller and state animal health officials, the herd owner agreed to herd testing in November 2013. North Dakota Department of Agriculture veterinarians conducted whole-herd testing in consultation with the USDA. All cows and heifers 6 months of age and older were tested using standard protocol of the caudal fold tuberculin (CFT) test in series with the comparative cervical tuberculin (CCT) test.

A 19-month-old pregnant heifer (ND1) was declared a reactor based on CCT testing. Upon necropsy, multiple micro abscesses were identified in a normal appearing mediastinal lymph node that was culture-positive for *M. bovis* at USDA's National Veterinary Services Laboratories (NVSL). Ten additional cows were CCT-negative and sent to slaughter in WI. Samples were collected from all 10 cows and although no gross lesions were identified, samples were submitted for culture at NVSL. Two of the 10 cows (ND2 and ND3) were determined to be infected with *M. bovis*. Microscopic granulomas in a lymph node that contained acid-fast bacteria from ND2 were PCR positive for MTBC but no *mycobacteria* were identified on culture. Representative, normal appearing lymph nodes from the head and thorax were submitted from ND3; these tissues were histologically negative for evidence of mycobacterial infection, but *M. bovis* was isolated from culture. Tissue from the post-mortem exam and the ear tags were DNA tested to confirm they were from the same animal and no errors were made during sampling, labeling, or processing. Over the course of testing, approximately 40 cattle were removed from the herd and no additional infected cattle identified.

**Figure 1:**
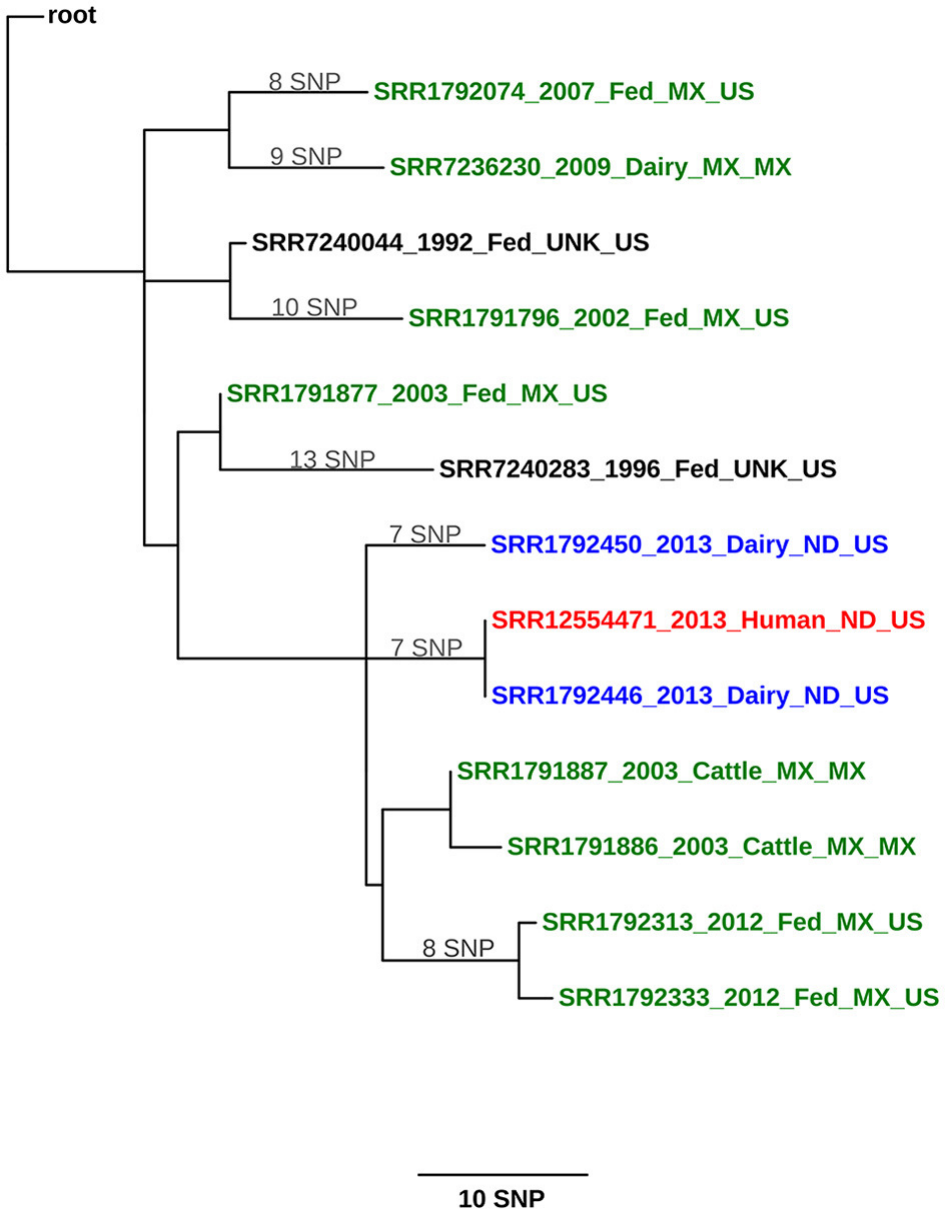
Maximum likelihood phylogenetic tree illustrating the genetic relationship between *M. bovis* isolates from a cattle herd in North Dakota (United States) and a human. The color key indicates the origin of the cattle from which *M. bovis* was isolated: green, Mexico; blue, United States; and black, cattle whose origin could not be traced (unknown). The human isolate is shown in red. Sequences are identified using the following syntax: NCBI SRA accession number_year of isolation_ production type (dairy, fed, cattle [unknown])_geographical origin of the animal (state, country or unknown)_country of detection. The scale bar represents a branch length of 10 SNPs. The tree is rooted to the reference genome *M. bovis* AF2122/97.

Whole genome sequencing was conducted and the infected pregnant heifer (ND1) had the identical strain to the dairy employee with active TB disease (Figure 1; see Supplementary File 1 for SNP table). The isolate from ND3 had seven single nucleotide polymorphisms (SNPs) compared with the isolates from ND1 and the case-patient. *Mycobacterium bovis* was not detected in the beef cattle. The infected dairy cattle were born and raised on the dairy operation. Two herds with fence line contact were tested and no infected animals were detected. Additionally, surveillance was conducted on barn cats, wild rodents, and hunter harvested deer with no disease detected. North Dakota Game and Fish Department conducted surveillance in the fall of 2014 on hunter-harvested deer and no lesions were identified. After a thorough investigation, no other possible sources of *M. bovis* were found.

### Case 2: Wisconsin Dairy Herd

Similar to the identification of the North Dakota herd, the Wisconsin Department of Agriculture, Trade and Consumer Protection (DATCP) was contacted in late April 2015 by the Wisconsin Department of Health Services (WIDHS) about a case-patient with TB disease that worked on a dairy from January to March 2015. The case-patient reportedly became ill in March 2015 before seeking medical care in early April. The case-patient presented in the emergency room with night sweats, cough, and fever. Sputum smears were positive for acid fast bacteria and nucleic acid amplification test (NAAT) was positive for MTBC. Culture revealed the case-patient was infected with *M. bovis*. The case-patient was placed in respiratory isolation and started on a standard four-drug regimen (45). After drug susceptibility testing was complete and pyrazinamide (PZA) resistance detected, PZA was discontinued. Due to severe cavitary disease, the case-patient was treated with TB medications for a full year, with directly observed therapy for the entire course. The case-patient was released from isolation in July 2015 when determined to no longer be contagious. The individual is believed to have become infected while previously living in a Latin-American country where *M. bovis* infections of humans and cattle are prevalent. Public health conducted a routine contact investigation that included TB risk assessment and testing of close contacts to the patient. No additional cases of infectious TB were identified in household or farm employee contacts.

In May 2015, DATCP conducted herd testing, in accordance with USDA guidance, of the 1,500-head herd. Like the ND herd, all cows and heifers 6 months of age and older were testing using the CFT test and CCT-test in series, with no infected animals detected. A single CCT-suspect cow from this first test was euthanized and necropsied with no lesions identified. In September 2015, the herd was tested a second time and was again test negative for bTB with no CCT-positive animals detected. In September 2018, slaughter plant surveillance detected tuberculosis in a carcass from a cow that traced back to this herd, and *M. bovis* was isolated. DATCP conducted another round of whole-herd testing using the CFT and CCT tests in series beginning in October 2018. Seven infected cows were identified during this initial test based on culture of *M. bovis*. Two additional infected cattle were detected during herd testing in March 2019, one infected cow was identified at slaughter in April 2020, and one cow was detected following herd testing in June 2020. Since the detection of the herd as infected, more than 1,500 cows have been examined for bTB by either necropsy or slaughter surveillance. One of the infected cows was test-negative and detected at slaughter. Additionally, surveillance was conducted on wildlife surrounding the premises and included white tailed deer (*n* = 232), raccoons (*n* = 10), and opossums (*n* = 6). One dairy that had heifers housed on the same premises as heifers from the affected dairy including fence line contact will be tested a total of three annual tests (2/3 have been completed and were negative).

**Figure 2:**
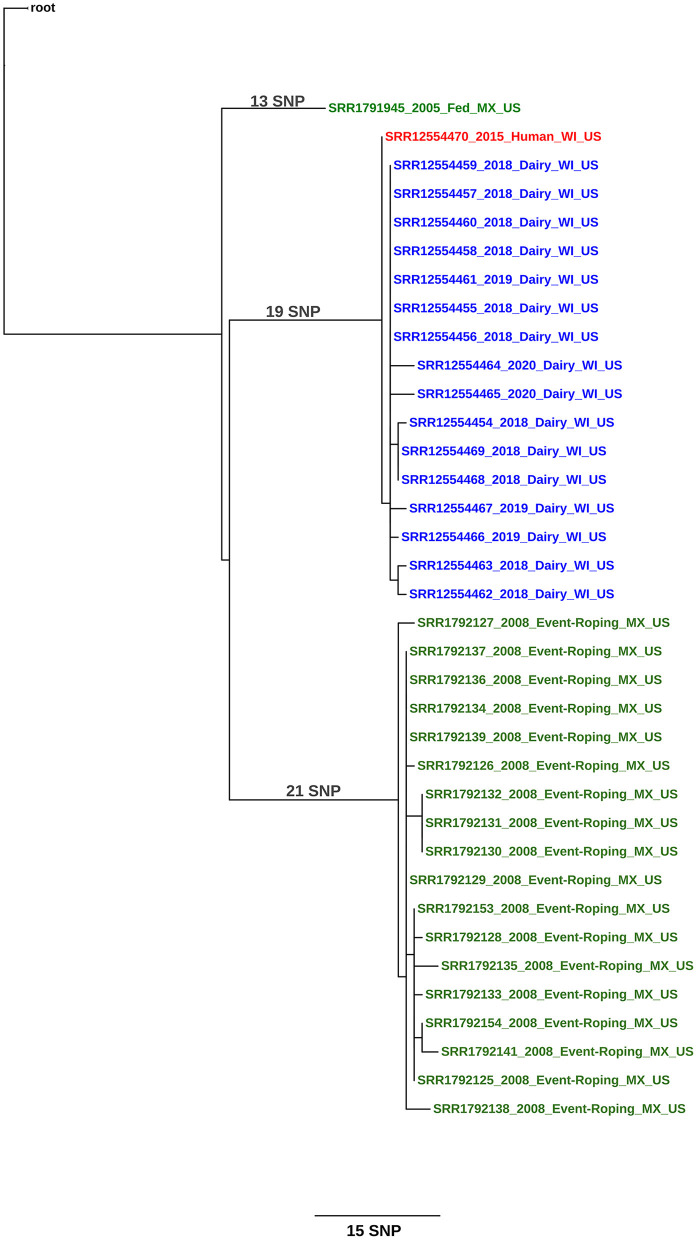
Maximum likelihood phylogenetic tree illustrating the genetic relationship between *M. bovis* isolates a herd in Wisconsin (United States) and a human. The color key indicates the origin of the cattle from which *M. bovis* was isolated: green, Mexico and blue, United States. The human isolate is shown in red. Sequences are identified using the following syntax: NCBI SRA accession number_year of isolation_ production type (dairy, fed, event-roping)_geographical origin of the animal (state, country)_country of detection. The scale bar represents a branch length of 15 SNPs. The tree is rooted to the reference genome *M. bovis* AF2122/97.

At the time of this writing, the herd has had a total of 12 cows, including the slaughter case, detected as infected with *M. bovis*; and the isolates were within a 1–4 SNP difference from the human isolate (Figure 2; see Supplementary File 1 for SNP table). The public health follow-up with farm employees continues until the farm is released from quarantine. Testing of source herds for purchased cattle added to the Wisconsin herd were not conducted since the epidemiologic investigation and WGS supported the human case-patient as the source of introduction into the herd.

### Case 3: Texas Dairy Heifer

In early 2018, a 1-day-old heifer calf from a dairy in New Mexico was transported to Texas where import regulations require post-import TB testing of cattle younger than 2 months of age that move into the state. The heifer was raised at a facility in Texas and was TB test positive in April 2018 at approximately 4 months of age. The heifer was euthanized and necropsied in late April 2018, and tissue samples were sent to NVSL for additional TB testing. There were no gross or microscopic lesions suggestive of MTBC infection. *Mycobacterium tuberculosis* was cultured from the retropharyngeal lymph node collected at necropsy and results were reported in June 2018. The heifer calf isolate grouped with sublineage 4.2.2 (43) and is in NCBI as accession SRR12481506.

The Texas Department of State Health Services (DSHS) conducted a source case investigation to determine if an infectious TB suspect had been identified by public health; however, a source case was not identified. Personnel from DSHS discussed the situation with dairy management, and a dairy employee was identified who exhibited signs and symptoms of TB but was no longer employed at the dairy. Whole genome sequencing of human cases was just being implemented by the Centers of Disease Control and Prevention, and consequently, the previous human cases in this area had not been sequenced. However, both the calf and three adults in the region that were diagnosed in 2017 and 2018 (46) had a matching, rather rare spoligotype for Texas, with an octal code 007000024000000. No employees were tested, but DSHS conducted symptom screening and provided education for the current employees. We were unable to retrospectively obtain WGS from any of the Texas human cases to include in this report.

## Discussion

The three cases presented here are the first formal reports of MTBC transmission from humans to cattle in the United States since 1968 (47). In both the North Dakota and Wisconsin cases, the human TB case-patients were exposed to cattle on the operation prior to or at the time bTB was discovered in the cattle. In the North Dakota case, bTB was detected in three dairy cattle within 2 months after the human was diagnosed; in the Wisconsin herd, it was 3 years between the human TB detection and the herd detection.

When bTB-affected herds in the United States are identified, state, and federal animal health agencies conduct epidemiologic investigations to determine the probable source of disease and trace any animals that left the herd to control disease spread. Historically, in the United States the most common sources for bTB infection in cattle have been other infected cattle. The exception to this is in several counties in Michigan where bTB is endemic in the wild white-tailed deer population. Investigations in Michigan have identified deer as the most common source of infection in cattle (48). Animal health authorities conduct interviews with herd owners to determine all potential routes of exposure, including sources of all purchased cattle over a multiyear period and any contact with cattle from other operations, including fence-line contact. These exposures are investigated, and all cattle contacts are tested to determine their bTB status per Bovine tuberculosis eradication uniform methods and rules (49). Additionally, most adult cattle that leave dairies undergo inspection at slaughter for bTB, which is the method by which most infected herds are identified.

Inspection of carcasses at slaughter, or slaughter surveillance has been an effective method of detecting bTB in cattle in the United States. This is highlighted by the WI herd being detected by slaughter surveillance and subsequently, having a test-negative, infected cow that was detected at slaughter. Both the ND and WI herds sent animals to slaughter prior to bTB detection and they continue to do so on a routine basis, providing additional surveillance for bTB. Although infected animals can be missed with slaughter surveillance, 47% of bTB affected herds are detected through slaughter surveillance, excluding herds in the endemic are of Michigan and a localized outbreak in Minnesota (17, 50).

The results of the North Dakota investigation suggest it is highly unlikely that cattle in the herd could have transmitted *M. bovis* to the human. The progression of disease in ND1 appeared to be relatively recent given the finding of *M. bovis* in a single mediastinal lymph node. In the Wisconsin case, the case-patient was diagnosed with TB within 2–3 months of employment. Although *M. bovis* infections in humans have been reported to progress from infection to clinical disease within a few months, more often this progression in humans takes much longer (51, 52). Most of the knowledge of TB in humans is based on infection with *M. tuberculosis* but it might not be the same for *M. bovis* infections.

Additionally, the ND patient's housemates were all tested and considered as LTBI, while the farm family were all negative for bTB. If the cattle were the source of the human infection, one would expect the farm family members to be exposed and potentially infected. The housemates of the patient were exposed to the patient and so it was expected that some or all of them would be infected. None of the patient's housemates were actively shedding *M*. bovis and considered as LTBI.

The North Dakota dairy herd has been tested 9 times since 2013, with only the three infected cows detected at the first herd test, despite this additional testing. Only one of those three infected cows (ND1) had a small gross lesion associated with bTB infection. The WGS of the *M. bovis* strain from the North Dakota case-patient and ND1 were an exact match. Significant epidemiological evidence supports the transmission of *M. bovis* from the employee to the heifer. Evidence includes the degree of illness in the employee with active TB disease (e.g., lung cavitation); the very small lesion in the infected heifer, possibly indicating recent infection; the lack of movement of animals on or off the farm; and the fact that the employee was born in Mexico, a TB-endemic country, and returned annually. Based on the epidemiological and laboratory evidence, the human was considered the most likely source of disease in the cattle by both animal and public health officials investigating the case.

One of the three bTB-infected cattle, ND2 was PCR positive but culture negative, and no isolate was available for WGS. The last cow, ND3, had normal appearing lymph nodes that were histologically negative but culture positive. The WGS revealed 7 SNP changes from the case-patient and ND1 strain. It is difficult to explain this finding based on the current data but we believe the human patient was likely infected with this strain variation. Humans and cattle have been found to be infected with multiple strains of MTBC, so it is possible the human was infected with more than 1 strain but only 1 was isolated. Unless a more closely related strain is identified in the future, the source of infection for ND3 will remain unknown. Based on the small size of the lesions, however, it is unlikely that ND3 was shedding *M. bovis*. An evaluation of the phylogenetic tree (and Supplementary File 1, SNP Table) shows that both ND1 and ND3 share a common ancestor with other Mexican cattle in the region that the dairy worker was known to previously reside and visit on an annual basis. This provides further support that the initial exposure of the case-patient was likely in their home country.

The Wisconsin case report provides the strongest epidemiologic evidence for human-to-cattle transmission, as the case-patient was diagnosed 3 years before the herd was detected. The individual was a very recent addition to the dairy's workforce and was diagnosed with TB disease within 3 months of beginning employment. Collectively, this provides strong evidence that the case-patient's exposure to *M. bovis* occurred prior to employment on the dairy. All cattle having potential contact with the case-patient were tested 2 and 6 months after the employee was no longer working on the dairy, and bTB was not detected. It is highly unlikely that infection was present and circulating in the herd and transmitted from cattle to the case-patient. Further, the WGS from the infected cows had at least 1 SNP change difference from the human and most recent common ancestor, or root sequence, suggesting the cattle isolates were closely related, direct descendants of the human isolate.

Results of testing of other dairy employees and family members continue to be negative since the initial tests in 2015. If the dairy cattle were the source of infection, we would have expected other employees or family members to be infected. The public health investigators were adamant that the employee with TB disease was very sick at the time of diagnosis and could not have progressed to this stage of disease in two months. They were confident the employee was infected prior to entering the dairy's workforce.

Investigators also found no evidence suggesting latent infection in the Wisconsin cattle. Three of the 12 infected cows were purchased additions (WI2, WI3, WI4), while the remaining cows were born and raised on the operation. One of the purchased cows (WI2) was brought on the operation in January 2015, roughly the same time the case-patient began employment. This cow was test negative in May and September 2015 suggesting she was not infected at the time of introduction into the herd. In October 2018, WI2 was found to be infected. The other two purchased cows were brought into the herd in 2016 after the case-patient was no longer present. Their infections are consistent with cattle-to-cattle transmission within the herd. Other than these two cows, the remaining 10 infected cows were all test negative at least once during the 2015 testing.

For both the North Dakota and Wisconsin cases, WGS supports that the TB case-patients were infected in their home country, possibly through contact with infected cattle or consumption of raw dairy products, and subsequently infected cattle on these farms after their disease became active. Others have reported similar scenarios where *M. bovis* infection makes the complete cycle from cow to human and back to cow (20).

While the Texas *M. tuberculosis* case lacks a confirmed case of human TB directly linking a human to the calf, the epidemiologic investigation and evidence of the matching spoligotype circulating in the local community strongly supports human to cattle transmission. Furthermore, a review of the literature found that, with one reported exception, humans are the direct source of *M. tuberculosis* infection for cattle. There is one report of a calf from an experimentally infected cow that was infected via colostrum or milk (53). To our knowledge, that has not been replicated in a naturally infected dairy. This is the first modern reported case *M. tuberculosis* infection in a U.S. bovine.

In addition to the three cases presented here, there have been at least 10 other U.S. bovine cases (i.e., affected herds) since 2009 that investigators were very suspicious of human-to-cattle *M. bovis* transmission as the source of introduction into these herds. These suspicions were based on both epidemiologic data collected, and WGS conducted by animal health officials, but all lacked the active, prospective identification of human cases.

Often, lesions are not present in young cattle diagnosed with *M. tuberculosis* (54–56). Although cattle without gross lesions are not considered a risk for transmission of disease to cattle or humans, *M. tuberculosis* has been found in cows' milk (57–59) and in granulomatous lesions from infected cattle, suggesting infected cattle may be a risk to other cattle and humans (23). Published reports suggest that humans are the primary source of *M. tuberculosis* infections in cattle with transmission occurring via the respiratory route (60). Although evidence for direct human-to-cattle transmission is not always present, *M. tuberculosis* has been found in cattle in multiple countries. Worldwide, human-to-cattle transmission of *M. tuberculosis* has been documented more frequently than *M. bovis*, likely due to the increased prevalence of *M. tuberculosis* in humans. More evaluations are needed to determine the importance of livestock in the transmission of *M. tuberculosis* between cattle and humans. The U.S has been characterizing MTBC isolates in livestock for over 40 years, and the last documented case of *M. tuberculosis* that occurred in U.S. livestock was a llama in 1991, associated with exotic animal trade (unpublished data).

Human-to-cattle transmission of *M. bovis* has infrequently been reported (20, 28, 47, 61–63) and there is only a single report of human-to-cattle *M. orygis* transmission (64). The finding of human-to-cattle transmission of *M. bovis* in the United States may be related to the increased risk of *M. bovis* infection among non-U.S.-born livestock workers compared to U.S.-born workers.

The prevalence of *M. bovis* in humans in the United States has declined from at least 10% of MTBC cases in 1900 to <2% of all MTBC cases in 2005 (65). Another publication demonstrated that human cases of *M. bovis* in the United States were more likely to be of Hispanic/Latino origin and born outside the United States (66). More recently, Scott et al. (67) reported human *M. bovis* prevalence in the United States with a similar prevalence of 1.3–1.6% of all MTBC cases, and a higher prevalence in children, Hispanics/Latinos, and females.

Although the number of human cases of MTBC infection in the United States has decreased about 90% since 1953 (68), some areas of the United States have reported an increase, especially along the southern U.S. border. A review of pediatric tuberculosis cases in San Diego, CA, from 1980 to 1997 revealed *M. bovis* was responsible for 10.8% of all TB cases and 33.9% of culture-positive cases (14). Hispanics represented 78.9% of the cases. More than half the *M. bovis* culture-positive case-patients (55.2%) had only extra-pulmonary bTB. This study highlights the concern of foodborne exposure via unpasteurized dairy products. Since dairy products appear to be the main source of human *M. bovis* infection in Mexico (17), efforts to eradicate bTB from the Mexican dairy industry must be strengthened to improve human and animal health in both the United States and Mexico.

Although the North Dakota and Wisconsin herds were most likely infected via the respiratory route, given active pulmonary disease in the workers, other publications suggest that extrapulmonary infections are more common with *M. bovis* infection. The authors concluded that the prevalence of extra-pulmonary disease in young, U.S.- or Mexican-born Hispanic/Latino populations suggested recent infection due to foodborne exposure (66). Extra-pulmonary disease was nine times more frequent among those with *M. bovis* than those with *M. tuberculosis*. Since transmission via urine has been reported in the literature in *M. bovis* cases (55), this possible extrapulmonary route of disease spread should be investigated when testing high-risk groups.

The median herd size of U.S. dairies has increased from 80 cows in 1987 to 1,300 cows in 2017 (69). The increase in the average size of dairy operations over the past few decades, in terms of the number of cows per herd, has resulted in dramatic needs for on-farm labor. Non-U.S.-born employees make up a significant portion of the workforce on U.S. dairy farms (70). A 2015 National Milk Producers Federation survey of 1,000 dairies (>50 cows) across the United States reported that 93% of operations hired outside labor and over 51% of employees were immigrants (71). A 2007 Wisconsin survey of dairy farms revealed that 40% of hired labor were immigrants; of these, 88.5% were from Mexico and most of the remainder of employees were from Central and South America (72). Based on the higher risk of MTBC infection in many non-U.S.-born employees compared with U.S. born workers, the Centers for Disease Control and Prevention recommends TB screening testing the high-risk groups (45).

Access to medical care can be challenging for non-U.S.-born employees. Often English language skills are limited, and employees may not have the documentation necessary to reside legally in the United States (71). A pilot project developed at the University of Wisconsin-Eau Claire School of Nursing was developed to immerse nursing students into Hispanic and rural culture. The focus of the program is to provide preventive healthcare and routine health screenings to a population that might not otherwise have access. Tuberculosis screening is included as one of the health screenings offered (73). Although this is a pilot project, it serves as a model that could be used in developing health-care programs that improve dairy employee health and safety.

The case reports presented here provide additional epidemiological support for human-to-cattle MTBC transmission and were largely the result of strong working relationships between animal health and public health in both North Dakota and Wisconsin. The communication and collaboration between animal health and public health officials to investigate cases of zoonotic diseases are crucial for gathering the information necessary to evaluate risk and identify effective preventive measures. While the actions of these two states can serve as a model, there are collaborative opportunities to establish additional best practices for issues important to both animal and human health.

Currently, the U.S. Cooperative State-Federal Bovine TB Eradication Program does not include mitigation strategies to address the risk of human introduction of MTBC into U.S. cattle herds, and these findings could change the paradigm of the program. A collaborative One Health approach is needed to address the health of the dairy workers and the animals. The U.S. government has defined One Health as “a collaborative, multisectoral, and transdisciplinary approach—working at the local, regional, national, and global levels—with the goal of achieving optimal health outcomes recognizing the interconnection between people, animals, plants, and their shared environment.” (74). In response, the U.S. dairy industry convened a multisectoral working group of state and federal animal and public health officials to address this challenge.

## Conclusion

This is this first published report using epidemiological and genotype evidence to establish human-to-cattle *M. bovis* transmission in the United States. This is also the first report of *M. tuberculosis* infection of cattle in the United States. In order to advance eradication of bTB in the U.S. cattle herd, the program must incorporate and address humans as another potential source of *M. bovis* or other MTBC species for cattle. This effort can only be achieved with a collaborative One Health approach that includes federal and state animal, public health, and wildlife agencies, livestock industries, producers, and healthcare workers and is focused on safeguarding both human and animal health.

## Data Availability Statement

The original contributions presented in the study are included in the article/Supplementary Material, further inquiries can be directed to the corresponding author/s.

## Ethics Statement

Ethical review and approval was not required for the study on human participants in accordance with the local legislation and institutional requirements. Written informed consent for participation was not required for this study in accordance with the national legislation and the institutional requirements. Ethical review and approval was not required for the animal study because this is a case series involving client owned cattle and is not a research project. Written informed consent for participation was not obtained from the owners because Regulatory procedures were performed that didn't necessarily require written consent. Written consent was obtained for animals removed based on results of regulatory testing.

## Author Contributions

JL, TD, CY, and SR-A contributed to the conception of the paper. EP, SG-B, RK, JT-K, BC, SK, DP, SR, WH, TT, and SR-A performed the investigations. JL, EP, TD, and SR-A wrote the first draft. CP constructed the phylogenetic trees. All authors contributed to manuscript revisions, read, and approved the submitted version.

## Conflict of Interest

The authors declare that the research was conducted in the absence of any commercial or financial relationships that could be construed as a potential conflict of interest.
